# Epigenetic markers in inflammation-related genes associated with mood disorder: a cross-sectional and longitudinal study in high-risk offspring of bipolar parents

**DOI:** 10.1186/s40345-019-0152-1

**Published:** 2019-08-06

**Authors:** Anne Duffy, Sarah M. Goodday, Charles Keown-Stoneman, Martina Scotti, Malosree Maitra, Corina Nagy, Julie Horrocks, Gustavo Turecki

**Affiliations:** 1 Division of Student Mental Health, Department of Psychiatry, Queen’s University, 146 Stuart Street, Kingston, ON Canada; 2 Department of Psychiatry, University of Oxford, Oxford, UK; 3 Dalla Lana School of Public Health, University of Toronto, Toronto, ON Canada; 4 McGill Group for Suicide Studies, Department of Psychiatry, Douglas Institute, McGill University, Montreal, QC Canada; 5 Department of Mathematics and Statistics, Guelph University, Guelph, ON Canada

**Keywords:** Bipolar disorder, High-risk offspring, Epigenetic markers, Methylation profiles, Longitudinal, Cross-sectional, Inflammatory candidate genes

## Abstract

Bipolar disorder is highly heritable and typically onsets in late adolescence or early adulthood. Evidence suggests that immune activation may be a mediating pathway between genetic predisposition and onset of mood disorders. Building on a prior study of mRNA and protein levels in high-risk offspring published in this Journal, we conducted a preliminary examination of methylation profiles in candidate immune genes from a subsample of well-characterized emergent adult (mean 20 years) offspring of bipolar parents from the Canadian Flourish high-risk cohort. Models were adjusted for variable age at DNA collection, sex and antidepressant and mood stabilizer use. On cross-sectional analysis, there was evidence of higher methylation rates for *BDNF*-*1* in high-risk offspring affected (n = 27) and unaffected (n = 23) for mood disorder compared to controls (n = 24) and higher methylation rates in affected high-risk offspring for *NR3C1* compared to controls. Longitudinal analyses (25 to 34 months) provided evidence of steeper decline in methylation rates in controls (n = 24) for *NR3C1* compared to affected (n = 15) and unaffected (n = 11) high-risk offspring and for *BDNF*-*2* compared to affected high-risk. There was insufficient evidence that changes in any of the candidate gene methylation rates were associated with illness recurrence in high-risk offspring. While preliminary, findings suggest that longitudinal investigation of epigenetic markers in well-characterized high-risk individuals over the peak period of risk may be informative to understand the emergence of bipolar disorder.

**Electronic supplementary material:**

The online version of this article (10.1186/s40345-019-0152-1) contains supplementary material, which is available to authorized users.

## Background

Collective research on the pathophysiology of bipolar disorder supports that immune activation may be a primary pathway mediating between genetic susceptibility and the onset of mood disorders, as well as influencing the observed changes in key neuroendocrine and neurotrophic systems (Raison and Miller 2013). Substantial evidence supports this theory including findings of elevated levels of peripheral inflammatory markers in depressed compared to non-depressed individuals and resolution of the inflammatory balance with successful treatment (Wiedlocha et al. 2018; Kohler et al. 2018). Further, changes in mRNA expression in pro-inflammatory genes in adult bipolar patients and their high-risk offspring have been reported, especially those with a past history of depression or who went on to develop depression (Padmos et al. 2008). While immune activation is neither necessary nor sufficient to explain the onset of mood disorders, it has been proposed that a genetically mediated vulnerability in the immune system may be amplified through interactions with other risk factors in high-risk individuals, including altered sensitivity of the glucocorticoid receptor and reduced BDNF production, culminating in illness onset (Raison and Miller 2013).

While heritability of bipolar disorder is estimated at around 85%, linkage, genome-wide association studies and polygenic risk profiles have not as yet been able to explain a significant proportion of the illness risk (Schulze et al. 2014; Gershon et al. 2011). However, epigenetic mechanisms or functional changes to the genome related to risk exposures, might provide an important biological mechanism that explains some of this missing heritability through gene-by-environment interactions characteristic of complex illnesses such as depression and bipolar disorder (Petronis 2010; Fiori et al. 2018).

In an earlier manuscript, we reported findings from a preliminary cross-sectional study of DNA variants, mRNA expression and protein levels in candidate immune and neurotrophic markers from plasma collected in prospectively assessed offspring of well-characterized bipolar parents from the Canadian Flourish high-risk study (Duffy et al. 2014a). Our findings supported that there may be identifiable differences in mRNA expression and protein levels (i.e., Il-6 and BDNF) in candidate inflammatory markers moderated by genetic variants (i.e., BDNF) in high-risk offspring compared to controls. Further, that changes in these peripheral markers (i.e., IL-6 and BDNF) appear to be associated with the emergent course of bipolar disorder.

Building on these earlier findings, here we report on a pilot study to explore DNA methylation changes in candidate genes associated with immune activation and the onset of mood disorders in a subset of subjects enrolled in the ongoing prospective Flourish Canadian high-risk offspring of bipolar parent study (Duffy et al. 2014b). Specific aims were to: (i) explore differentially methylated regions (DMRs) between three study groups: high-risk offspring unaffected or affected for lifetime mood disorder and unaffected controls and (ii) to explore longitudinal DMRs in these study groups.

## Methods

### Participants

High-risk (HR) offspring and controls with salivary DNA samples were selected from agreeable participants already enrolled in the Canadian Flourish high-risk offspring study which has been described in detail elsewhere (Duffy et al. 2014b, 2018). Briefly, high-risk offspring were identified from families with one parent with confirmed BD (other parent well) and controls from families in which both parents were confirmed to be free from major psychiatric disorder at the time of recruitment on the basis of clinical research (SADS-L) interviews. Study offspring were clinically assessed (initially blindly) on average annually using KSADS-PL/SADS-L interviews conducted by a research psychiatrist. DSM-IV diagnoses were based on blind consensus review using best estimate diagnostic procedure. At routine research visits repeated salivary samples were collected using free drool method in Oragene Genotek DNA kits. All participants were assessed at the time of collection to be well or in good quality of remission based on clinical research interview as part of the high-risk study protocol. Pharmacological treatment was routinely captured during clinical research assessments and defined as any prescribed use of antidepressant or mood stabilizer (i.e. lithium, antipsychotic, and/or anticonvulsant).

This pilot study was funded to analyze samples from a maximum of 25 participants in each study group at one time point in the cross-sectional and two time points in the longitudinal study. Participants represent a subset of consenting offspring from the larger Flourish cohort who provided salivary DNA samples and were selected into cross-sectional and longitudinal analysis subgroups based on inclusion criteria to ensure comparability between groups. We attempted to frequency match on age and sex to our best ability given the available samples. Subjects with a recent history (i.e., within 3 months) of substance use disorder or exposure to anti-inflammatory medication were excluded from enrolment for this sub-study.

The cross-sectional analysis included an *affected* HR offspring group who met criteria for a DSM-IV lifetime diagnosis of a major mood disorder (i.e., major depression single/recurrent, BD II, BD I, or BDNOS), and an *unaffected* HR offspring group with no lifetime history of any diagnosable major mood disorder; and a control group of offspring of well parents who themselves were unaffected for any diagnosable psychiatric disorder. Participants were selected for the longitudinal analysis if they had at least 2 repeated salivary DNA samples and for the HR affected group had at least 1 major mood episode in between samples, while criteria for unaffected and control groups were defined as the same as in the cross-sectional analysis. This study was approved by the Independent Review Ethics Board in Ottawa.

### Methylation assay

We used targeted bisulfite sequencing (Chen et al. 2017) to assess the DNA methylation level at specific genomic sites using DNA isolated from the saliva of eligible HR offspring and control subjects. Given the limited budget and preliminary nature of this study, we focused on 6 amplicons from the 5 candidate genes having the highest probability of biological effect on immune and related endocrine and neurotrophic pathways: *IL*-*1β*, *IL*-*6*, *TNF*-*α,* glucocorticoid receptor (*NR3C1*), *BDNF (BDNF*-*1* corresponding to promoter 1 *and BDNF*-*2* corresponding to promoter 4).

Briefly, genomic DNA was extracted from lysed saliva stored in Oragene DNA tubes containing DNAse inhibitors (DNA genotek, Ontario, Canada), following the manufacturer protocol. DNA was measured using the NanoDrop 2000 and 1–2 μg of DNA was used for bisulfite conversion with the Epitect plus DNA bisulfite kit (Qiagen, Venlo, Netherlands), following the manufacturer’s instructions. Primers were designed for each gene of interest using methyl Primer Express v1.0 (Applied Biosystems), targeting amplicon lengths of ~ 500 bp. Amplicons were fitted with adaptors, sequencing primer, sample specific indices, and deep sequenced to reach, in most cases, 1000× coverage. Reads produced by the sequencer were trimmed to remove low-quality bases and adapters. Once cleaned, the reads were aligned to bisulfite-converted versions of each strands of the reference genome. Methylation was then extracted by identifying relevant base substitutions at cytosine positions of the original reference genome and a percentage change was calculated from the reads aligning to the given cytosine position. Sequencing was not successful for *TNF*-*α* and thus dropped from the study.

### Statistical analysis

For the cross-sectional analyses linear general estimating equations (GEE) were used to account for repeated measures within families. Models were adjusted for variable age at DNA collection, and sex. Based on assessment of the model residuals, log transformations of the methylation rates were necessary to satisfy the normality model assumptions.

For the longitudinal analyses, linear mixed effects regression models using compound symmetry covariance to adjust for repeated measures within subjects were used. As with the cross-sectional analyses, the outcome in each model was the log transformed methylation rate of the genes of interest. Models were adjusted for variable age at DNA collection, sex and antidepressant and mood stabilizer use between samples (see Additional file 1: Additional statistical model information for further details). CpGs were only included in the statistical analysis if they had a minimum of 5 reads per site and any samples where coverage was sparse were also excluded from the analysis. All statistical analyses were conducted using SAS version 9.4 for Windows 64bit (Ins SI 2017).

## Results

The cross-sectional cohort included 74 subjects (27 affected HR, 23 unaffected HR, and 24 unaffected controls). The longitudinal cohort included 50 subjects (15 affected HR, 11 unaffected HR, and 24 unaffected controls). The mean age at first DNA sample was 21.1 years [4.7 standard deviation (SD)] in the cross-sectional cohort, and 20.5 years (4.4 SD) in the longitudinal cohort. Sex ratio and SES were comparable between study groups in both the cross-sectional and the longitudinal cohorts. The mean time between Time 1 and Time 2 samples was 25 months in controls, 26 months in affected HR offspring and 34 months in unaffected HR offspring (p = 0.0589), (see Additional file 1: Tables S1, S2 for more detailed group comparisons). Furthermore, most HR affected had a single episode between T1 and T2 samples of depressive polarity that on average lasted 17 months (see Additional file 1: Table S2).

### Cross-sectional analysis of methylation profiles

**Fig. 1:**
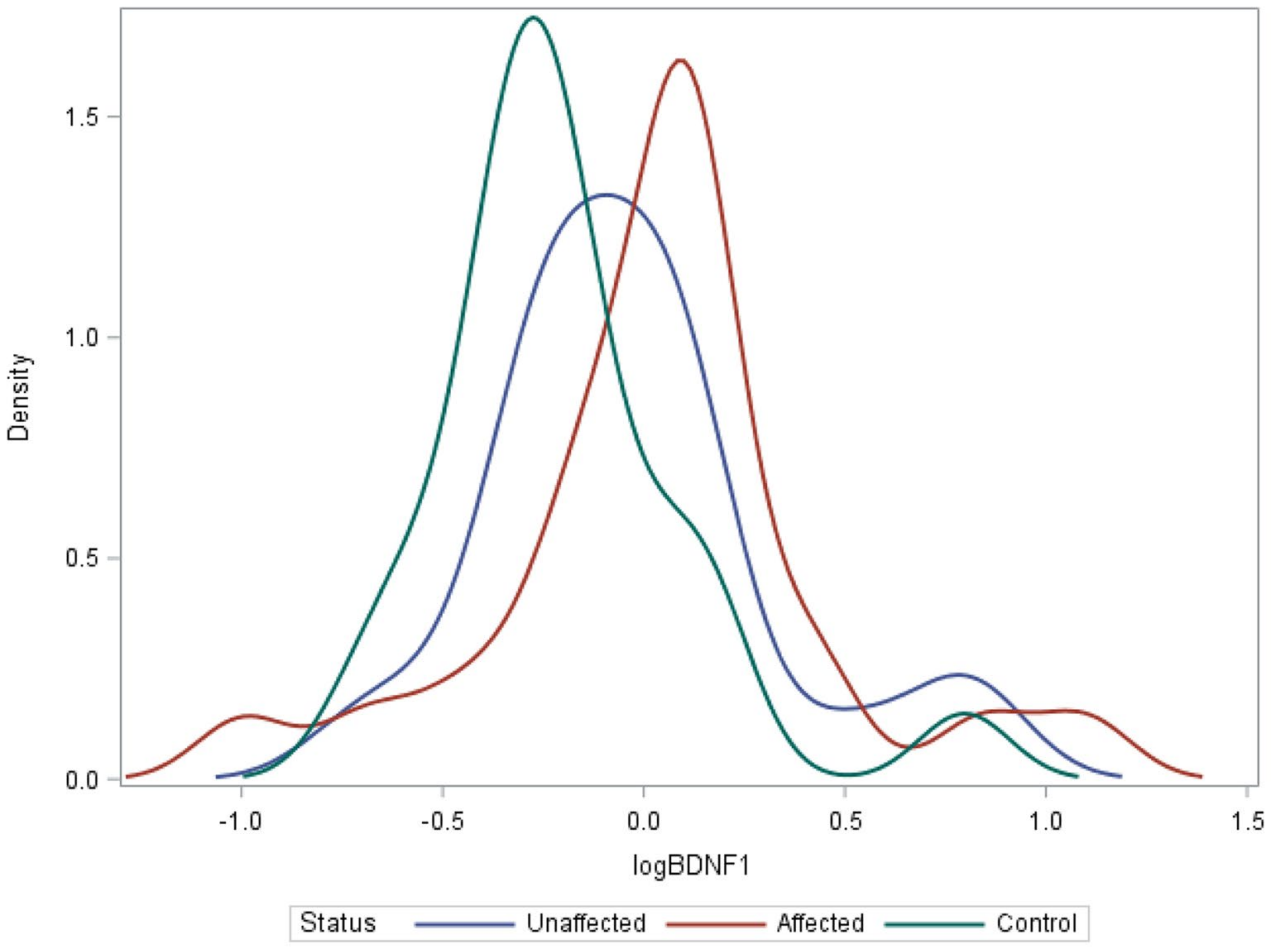
Kernel smoothed log *BDNF*-*1* methylation profiles in cross-sectional analysis between affected and unaffected high-risk (HR) offspring and controls. Estimated distribution of log BDNF-1 methylation rates. After adjustment, there was evidence that the mean of controls (green) is different from the mean of unaffected HR (blue) (p = 0.0385, z-value = − 2.07), and affected HR (red) (p = 0.0230. z-value = − 2.27). There was no evidence that the mean of affected HR (red) was different from unaffected HR (blue) (p = 0.9645, z-value = − 0.04)

**Fig. 2:**
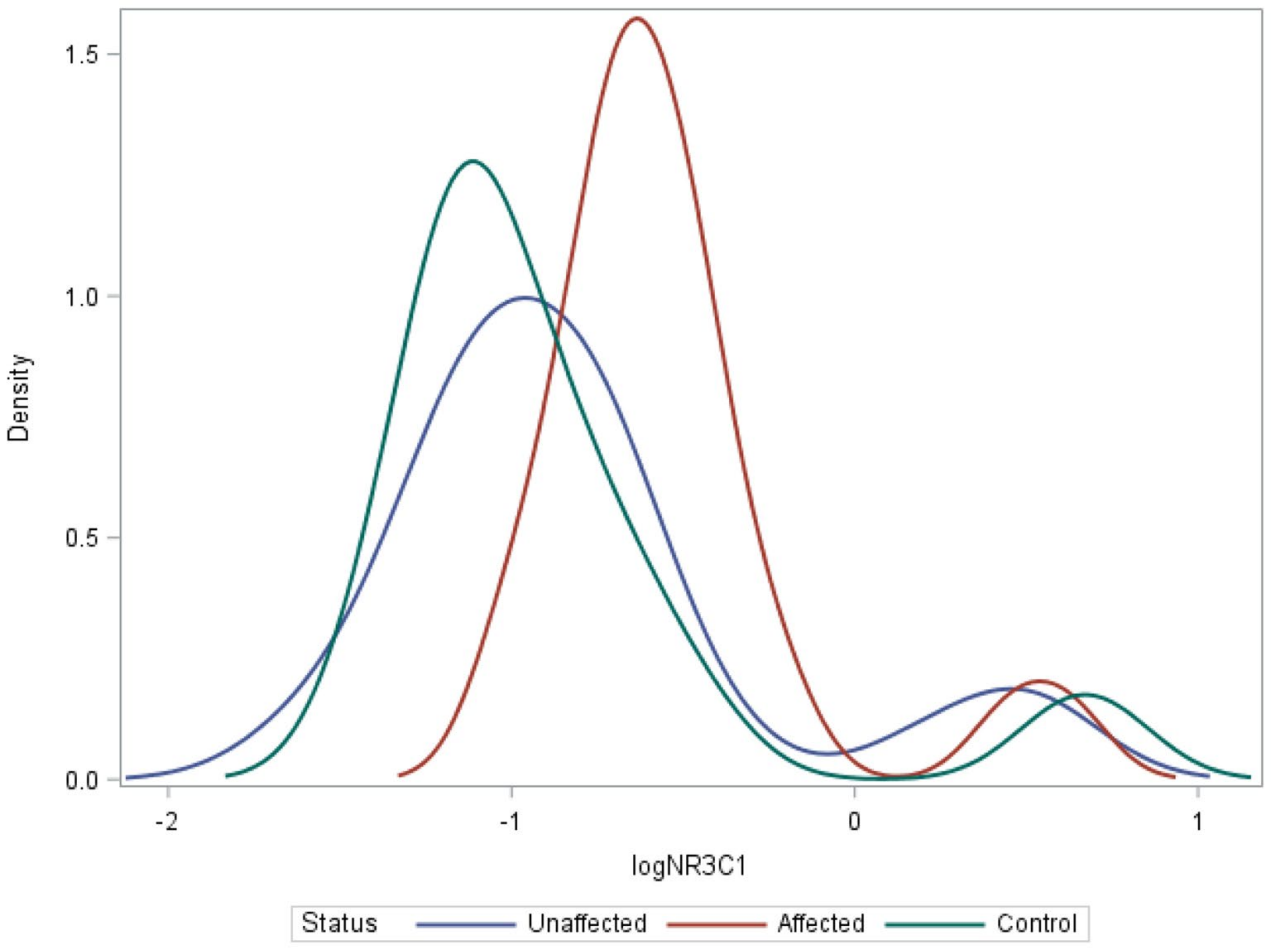
Kernel smoothed log *NR3C1* methylation profiles in cross-sectional analysis between affected and unaffected high-risk (HR) offspring and controls. Estimated distribution of log NR3C1 methylation rates. After adjustment, there was evidence that the mean of controls (green) is different from the mean of affected HR (red) (p = 0.0396, z-value = − 2.06), but not unaffected HR (blue) (p = 0.3094, z-value = − 1.02). There was also no evidence that the mean of affected (red) was different from unaffected HR (blue) (p = 0.5597, z-value = 0.58)

There was no evidence of a difference in mean log methylation values between groups for *IL*-*1β*, *IL*-*6* (Table 1) (Additional file 1: Figures S2, S3). In comparison to controls, there was evidence of higher *BDNF*-*1* methylation in unaffected and affected HR subjects (β^a^ − 0.2057; p = 0.0385 and β^a^ − 0.2004, p = 0.0230, respectively; Table 1; Fig. 1). There was also evidence of higher mean log *NR3C1* methylation in affected HR offspring compared to controls (β^a^ − 0.2466, p = 0.0396; Table 1; Fig. 2). Mean log *BDNF*-2 methylation was higher in affected HR offspring compared to unaffected HR offspring but this fell short of statistical significance after adjustments (β^a^ 0.2081, p = 0.0567) (Additional file 1: Figure S1). There was no evidence of differences in methylation rates between affected HR and unaffected HR for all other candidate genes.

**Table 1:** Cross-sectional and longitudinal differences in candidate gene methylation rates between study groups

Candidate genes	Control vs HR unaff			Control vs HR aff			HR aff vs HR unaff		
	β^a^	*p* value	z-value	β^a^	p-value	z-value	β^a^	p-value	z-value
Cross-sectional differences in methylation rates									
*BDNF*-*1*	− 0.2057	*0.0385*	− 2.07	− 0.2004	*0.0230*	− 2.27	− 0.0053	0.9645	− 0.04
*BDNF*-*2*	0.1715	0.1070	1.61	− 0.0366	0.7068	− 0.38	0.2081	0.0567	1.91
*IL*-*1β*	− 0.0593	0.6414	− 0.47	− 0.1433	0.2203	− 1.23	0.0840	0.5419	0.61
*IL*-*6*	0.0594	0.6272	0.49	0.0153	0.9096	1.11	0.0441	0.7287	0.36
*NR3C1*	− 0.1528	0.3094	− 1.02	− 0.2466	*0.0396*	− 2.06	0.0938	0.5597	0.58

Candidate genes	Control vs HR unaff			Control vs HR aff			HR aff vs HR unaff		
	β^b^	p-value	t-value	β^b^	p-value	t-value	β^b^	p-value	t-value
Longitudinal changes in methylation rates from T1 to T2									
*BDNF*-*1*	− 0.013	0.3230	− 1.00	0.001	0.9160	0.11	− 0.014	0.3521	− 0.94
*BDNF*-*2*	− 0.014	0.0624	− 1.91	− 0.021	*0.0140*	− 2.55	0.007	0.4384	0.78
*IL*-*1β*	0.001	0.9344	0.08	− 0.011	0.2358	− 1.20	0.012	0.2315	1.21
*IL*-*6*	− 0.002	0.8424	− 0.20	− 0.009	0.3262	− 0.99	0.007	0.4484	0.76
*NR3C1*	− 0.020	*0.0025*	− 3.20	− 0.023	*0.0019*	− 3.30	0.003	0.6897	0.40

Significant p values are in italic

^a^Beta coefficient for the estimated differences between groups in a multivariable mixed model including main effects for group, age, sex, and lifetime antidepressant and mood stabilizer use

^b^Beta coefficient from the interaction product term between time and group in a multivariable mixed model including main effects for group, age, sex, antidepressant and mood stabilizer use between samples

### Longitudinal analysis of methylation profiles

**Fig. 3:**
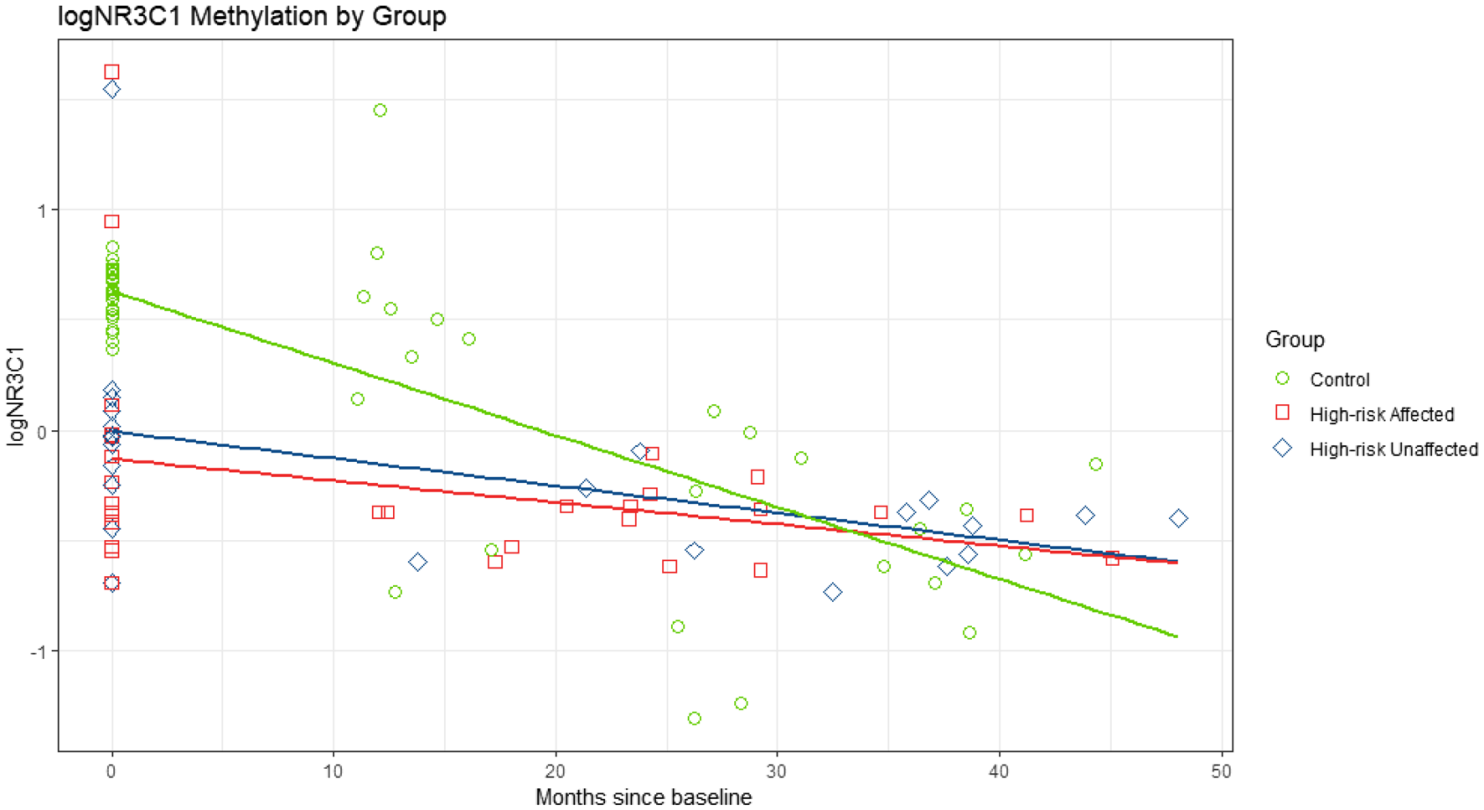
Log NR3C1 methylation percentage by study group from the longitudinal model between Time 1 and tIme 2. Estimated longitudinal profiles of log NR3C1 methylation rates. After adjustment, there was evidence that the rate of decrease in controls (green) is steeper than the rate of decrease of unaffected HR (blue) (p = 0.0025, t-value = − 3.20), and affected HR (red) (p = 0.0019, t-value = − 3.30). There was no evidence that the change over time in affected HR (red) was different from unaffected HR (blue) (p = 0.6897, t-value = 0.40)

**Fig. 4:**
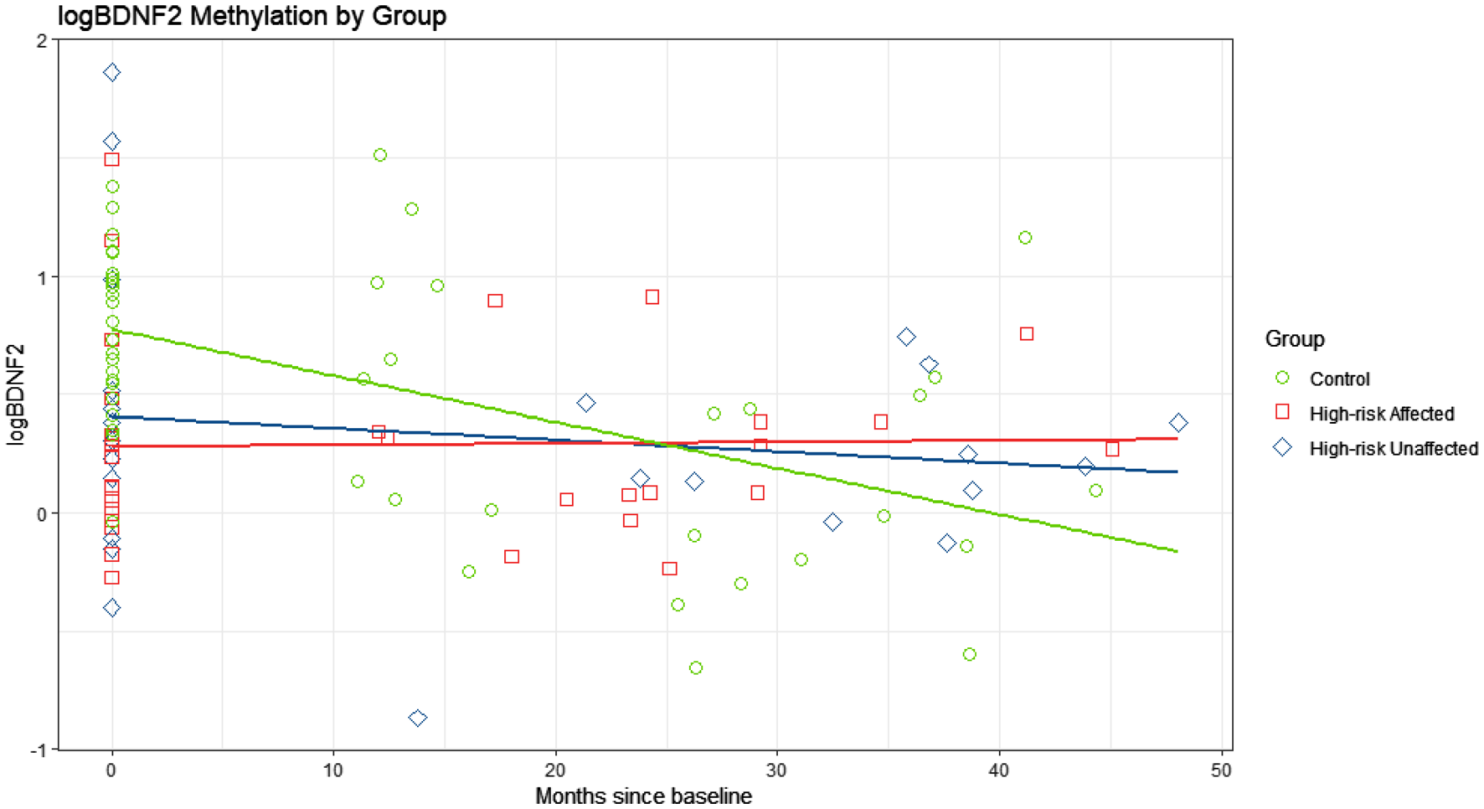
Log BDNF-2 methylation percentage by study group from the longitudinal model between Time 1 and Time 2. Estimated longitudinal profiles of log BDNF-2 methylation rates. After adjustment, there was evidence that the rate of decrease in controls (green) is steeper than the rate of decrease of affected HR (red) (p = 0.0140, t-value = − 2.55), and marginally steeper than in unaffected HR (blue) (p = 0.0624, t-value = − 1.91). There was no evidence that the change over time of affected HR (red) was different from unaffected HR (blue) (p = 0.4384, t-value = 0.78)

There was evidence that methylation profiles for *BDNF*-*1*, *BDNF*-*2*, *IL*-*1β*, and *NR3C1* changed over time (p = 0.0012; p = 0.0367; p = 0.0042; p < 0.0001) with an estimated decrease in all study groups except *BDNF*-*2* affected offspring, while *IL*-*6* remained stable (Figs. 3, 4; Additional file 1: Figures S4–S6). For all examined gene profiles, there was insufficient evidence that changes in methylation rates within affected HR offspring experiencing a major mood episode between Time 1 and Time 2 samples were different than changes in unaffected HR. For *BDNF*-*2*, there was evidence of a steeper decline in in controls compared to affected HR offspring (β^b^ − 0.021, p = 0.0140). Similarly, for *NR3C1*, there was evidence of a steeper decline for controls versus HR unaffected (β^b^ − 0.020, p = 0.0025) and for controls versus affected HR offspring (β^b^ − 0.023, p = 0.0019) (Table 1; Figs. 3, 4). In all models tested, there was no evidence of an effect of antidepressant or mood stabilizer exposure on methylation rates. Unadjusted models yielded similar patterns and levels of significance.

## Discussion

The purpose of this preliminary study was to explore methylation profiles in candidate inflammatory-related genes in adolescent and emergent adults at confirmed familial risk of developing bipolar spectrum disorders. Findings suggest that there may be detectable differences in methylation profiles between HR offspring and low risk controls, although the nature and clinical importance of these differences remains unclear. Specifically, we found higher mean methylation percentages in *BDNF*-*1* in HR offspring affected and unaffected for lifetime major mood disorders compared to unaffected low risk controls. Furthermore, there was higher mean methylation percentages in HR offspring affected for mood disorder compared to controls for *NR3C1.*

In the longitudinal study, we found evidence of a general decline in methylation rates in most candidate genes over time in both HR offspring and controls. The rate of decline in methylation percentages of *NR3C1* was greater in controls compared to HR affected and unaffected offspring. Similarly, the rate of decline in methylation percentages of *BDNF*-*2* was greater in controls compared to affected HR offspring and there was marginal evidence of a difference compared to unaffected HR offspring. There was no evidence that changes in methylation rates over time in HR offspring were associated with increases in burden of illness. From the cross-sectional analysis there was no evidence of a difference in methylation rates in candidate genes between HR affected and HR unaffected study groups with the exception of marginal evidence of a difference in *BDNF*-*2.* Further, from the longitudinal analysis there was no evidence of a difference in change in methylation profiles over time between HR unaffected and HR affected (with an intervening episode of illness between Time 1 and Time 2).

These findings need to be interpreted with caution given several limitations. Results are based on small sample sizes and given the exploratory nature were unadjusted for multiple comparisons, although unadjusted higher-powered models showed similar patterns of findings across all candidate genes examined. Several other factors may have impacted methylation percentages such as early risk exposures not accounted for in this analysis. DNA methylation is binary, either existing or not, at a given CpG position. This project derives data from a homogenate of cells, therefore methylation values are represented as a percentage that depicts the proportion of cells garnering a particular methylated position. The low percentage of methylation identified in this study makes it difficult to assess the biological relevance of the described changes as they theoretically relate to a very small number of cells. However, it is important to consider that methylation is cell type specific and the low methylation profile identified here may be the result of cellular composition. In other words, the cell type or types of interest may have been underrepresented in the pool.

Technical limitations include the inability to separate cell types, or accounting for variable cellular composition between subjects (Theda et al. 2018). The validity of bisulfide amplicon sequencing is dependent on sequencing coverage, where areas of low coverage could result in false positives or negatives. In this study, any CpG with low coverage or, a poorly sequenced individual sample, were excluded from analyses. The majority of CpGs had well over 500-fold coverage, rendering high confidence results in spite of the very low number of cells presenting methylation at the targeted gene regions.

This study highlighted the importance of including a comparison group without the risk of interest in the longitudinal study as there may be normative changes in DNA methylation that accompany aging and ambient exposures. Moving forward, it would be important to include a larger sample of well-characterized high-risk subjects assessed repeatedly over the full risk period and accounting for the influence of other risk exposures, such as early trauma and cumulative life stress. Given limited resources to fund this preliminary study, we adopted a candidate gene approach. However, given the apparent genetic complexity of mood disorders, a whole genome approach would be more powerful. Further, to accurately evaluate variability in methylation profiles, a higher number of repeated samples for the analysis would be more informative. Further comparison of methylation profiles across different tissue samples (i.e., blood and saliva) and using buccal swabs rather than free drool saliva might be more informative (Theda et al. 2018).

In summary, epigenetic changes in gene function are thought to play an important role in bipolar illness onset in individuals at confirmed genetic risk. This study suggests that differences in methylation profiles may be a useful way of studying epigenetic changes associated with risk status. Moving forward what is needed are longitudinal studies of large populations of well-characterized high-risk individuals including fine-grained mapping of exposures to higher frequency repeated samplings. This study is an initial step showing the importance of considering normative changes in DNA methylation likely related to age and ambient exposures.

## Additional file

**Additional file 1.** Supplementary tables and figures.

## Data Availability

In accordance with this study’s research ethics approval, de-identified data may be accessed via request to and approval from the principle investigator Dr. Anne Duffy.

## Abbreviations

BD: bipolar disorder
*BDNF*: brain derived neurotrophic factor
DSM: Diagnostic and Statistical Manual for Mental Disorders
GEE: generalized estimating equations
HR: high-risk
*IL*: interleukin
KSADS-PL: kiddie schedule for affective disorders-present and lifetime version
SD: standard deviation
*TNF*-*α*: tumor necrosis factor-alpha
*NR3C1*: nuclear receptor subfamily 3 group C member 1
