# Supplementary material for: Epigenetic markers in inflammation-related genes associated with mood disorder: a cross-sectional and longitudinal study in high-risk offspring of bipolar parents
Source: Int J Bipolar Disord. 2019 Aug 6;7:17. doi: 10.1186/s40345-019-0152-1 (PMC6682840; doi:10.1186/s40345-019-0152-1)

# Table S1: Cross-sectional Study - Description of Sample

|  | High-risk  Affected  (N=27) | High-risk Unaffected  (N=23) | Control  (N=24) | p-value |
| --- | --- | --- | --- | --- |
| Age at DNA sample |  |  |  |  |
| Mean( SD) | 22.91 (4.35) | 19.61 (6.02) | 20.59 (2.65) | 0.0337^a^ |
|  |  |  |  |  |
| Sex n(%) |  |  |  |  |
| Male | 7 (25.9) | 7 (30.4) | 8 (33.3) | 0.8430^b^ |
| Female | 20 (74.1) | 16 (69.6) | 16 (66.7) |  |
|  |  |  |  |  |
| SES n(%) |  |  |  |  |
| 1 | 0 (0.0) | 0 (0.0) | 0 (0.0) | 0.2547^b^ |
| 2 | 0 (0.0) | 0 (0.0) | 3 (12.5) |  |
| 3 | 4 (14.8) | 2 (8.7) | 2 (8.3) |  |
| 4 | 8 (29.6) | 9 (39.1) | 9 (37.5) |  |
| 5 | 15 (55.6) | 12 (52.2) | 10 (41.7) |  |
|  |  |  |  |  |
| Lifetime major mood |  |  |  |  |
| Age of onset Mean (SD) | 17.10 (3.92) | - | - | - |
| Age of onset (min, max) | (7.08, 25.17) | - | - | - |
|  |  |  |  |  |
| Treated with an antidepressant^d^ n(%) |  |  |  |  |
| Yes | 18 (66.7) | 0 (0.0) | 1 (4.2) | <0.0001^b^ |
| No | 9 (33.3) | 23 (100.0) | 23 (95.8) |  |
|  |  |  |  |  |
| Treated with a mood stabilizer^e^ n(%) |  |  |  |  |
| Yes | 14 (53.8) | 0 (0.0) | 0 (0.0) | <0.0001^c^ |
| No | 12 (46.2) | 23 (100.0) | 24 (100.0) |  |

a ANOVA

b Chi-square test

c Fisher’s Exact Test

d Lifetime antidepressant exposure

e Lifetime lithium, antipsychotic, and/or anticonvulsant exposure

# Table S2: Longitudinal Study - Description of Sample

|  | High-risk  Affected  (N=15) | High-risk Unaffected  (N=11) | Control  (N=24) | p-value |
| --- | --- | --- | --- | --- |
| Age at first DNA sample |  |  |  |  |
| Mean (SD) | 22.25 (4.94) | 18.14 (5.79) | 20.27 (2.31) | 0.0512^a^ |
|  |  |  |  |  |
| Months between Time 1 & Time 2 DNA samples |  |  |  |  |
| Mean (SD) | 26.12 (9.24) | 33.94 (9.98) | 24.89 (11.12) | 0.0589^a^ |
| (min, max) | (12.02, 45.08) | (13.77, 48.07) | (11.10, 44.35) |  |
|  |  |  |  |  |
| Number of Episodes between samples n(%) |  |  |  |  |
| 1 | 13 (86.7) | - | - | - |
| 2 | 2 (13.3) | - | - |  |
|  |  |  |  |  |
| Polarity of episodes between samples n(%) |  |  |  |  |
| Hypomania | 2 (13.3) | - | - | - |
| Major Depression | 10 (66.7) | - | - |  |
| Depression NOS | 2 (13.3) | - | - |  |
| Mixed | 1 (6.7) | - | - |  |
|  |  |  |  |  |
| Duration of episodes (weeks) |  |  |  |  |
| Mean (SD) | 17.00 (20.07) | - | - | - |
| (min, max) | (1, 84) | - | - |  |
|  |  |  |  |  |
| Sex n(%) |  |  |  |  |
| Male | 4 (26.7) | 4 (36.4) | 12 (50.0) | 0.3376^b^ |
| Female | 11 (73.3) | 7 (63.6) | 12 (50.0) |  |
|  |  |  |  |  |
| SES n(%) |  |  |  |  |
| 1 | 0 (0.0) | 0 (0.0) | 0 (0.0) | 0.5821^c^ |
| 2 | 0 (0.0) | 0 (0.0) | 2 (8.3) |  |
| 3 | 3 (20.0) | 0 (0.0) | 2 (8.3) |  |
| 4 | 8 (53.3) | 6 (54.5) | 10 (41.7) |  |
| 5 | 4 (26.7) | 5 (45.5) | 10 (41.7) |  |
|  |  |  |  |  |
| Lifetime major mood |  |  |  |  |
| Age of onset Mean (SD) | 18.52 (3.79) | - | - | - |
| Age of onset (min, max) | (14.41, 25.17) | - | - |  |
|  |  |  |  |  |
| Treated with an antidepressant^d^ between samples n(%) |  |  |  |  |
| Yes | 3 (20.0) | 2 (18.2) | 0 (0.0) | 0.0348^c^ |
| No | 12 (80.0) | 9 (81.8) | 24 (100.0) |  |
|  |  |  |  |  |
| Treated with a mood stabilizer^e^ between samples n(%) |  |  |  |  |
| Yes | 3 (20.0) | 0 (0.0) | 0 (0.0) | 0.0316^c^ |
| No | 12 (80.0) | 11 (100.0) | 24 (100.0) |  |

a ANOVA

b Chi-square test

c Fisher’s Exact Test

d Antidepressant treatment between T1 and T2 samples

e Lithium, antipsychotic, and/or anticonvulsant treatment between T1 and T2 samples

**Additional statistical model information**

The fixed effects for the minimally-adjusted longitudinal models were of the following from:

$$E\left[ \log\left( \mathrm{methylatio}n_{ij} \right) \right]=\beta_{0}+\beta_{1}T_{ij}+\beta_{2}C_{i}+\beta_{3}A_{i}+\beta_{4}T_{ij}C_{i}+\beta_{5}T_{ij}A_{i},$$

and the fully-adjusted models were of the following form:

$$E\left[ \log\left( \mathrm{methylatio}n_{ij} \right) \right]=\beta_{0}+\beta_{1}T_{ij}+\beta_{2}C_{i}+\beta_{3}A_{i}+\beta_{4}T_{ij}C_{i}+\beta_{5}T_{ij}A_{i}$$

$$+\beta_{6}Age_{i}+\beta_{7}Sex_{i}+\beta_{8}AD_{i}+\beta_{9}{MS}_{i},$$

where,

- *T_ij_* = the number of months since baseline
- *A_i_* = 1 if subject *i* was affected at follow-up and 0 otherwise
- *C_i_* = 1 if subject *i* was in the control group and 0 otherwise
- *Age_i_* = the age of subject *i* at baseline
- *Sex_i_* = 1 if subject *i* is male and 0 if they are female
- *AD_i_* = 1 if subject *i* was treated with anti-depressants between baseline and follow-up and 0 otherwise
- *MS_i_* = 1 if subject *i* was treated with mood stabilizers between baseline and follow-up and 0 otherwis

**Figure S1.** Cross-sectional Study - *BDNF-2* Methylation profiles


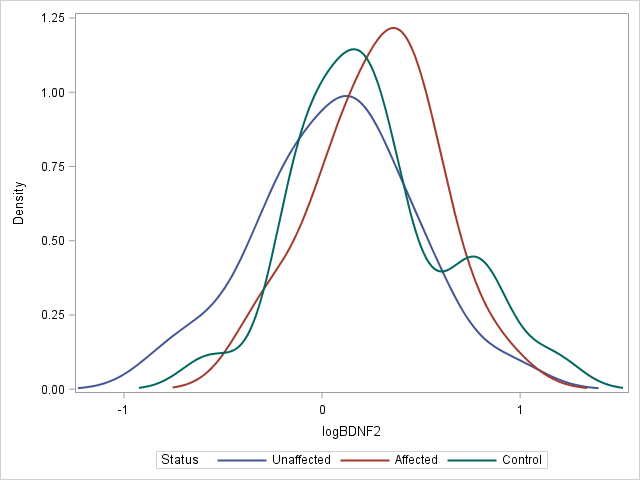


**Figure S2.** Cross-sectional Study - *IL-6* Methylation profiles


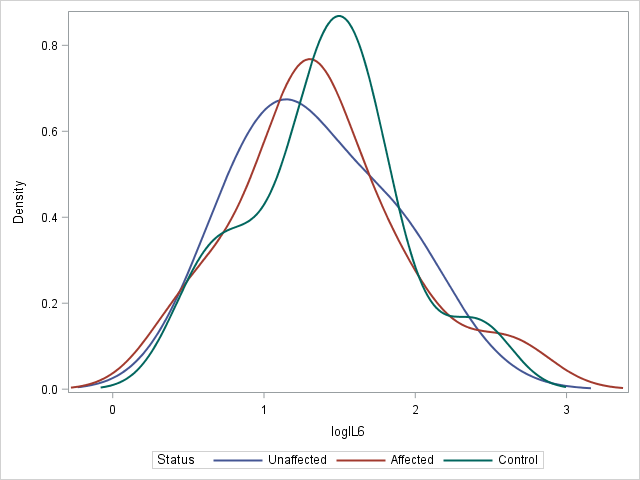


**Figure S3.** Cross-sectional Study - *IL1B* Methylation profiles


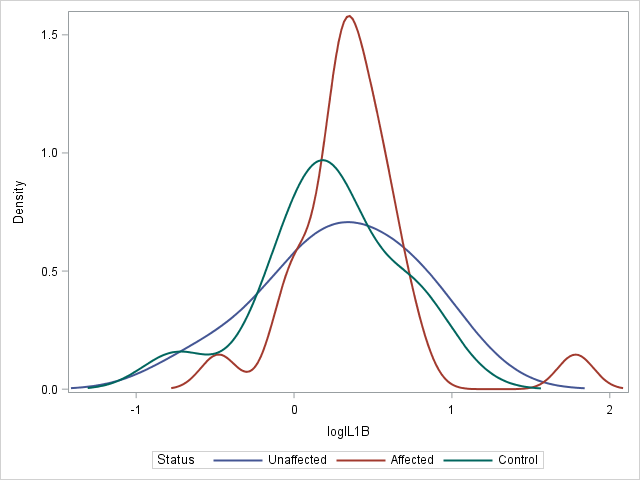


**Figure S4.** Longitudinal Study- *BDNF-1* methylation profiles


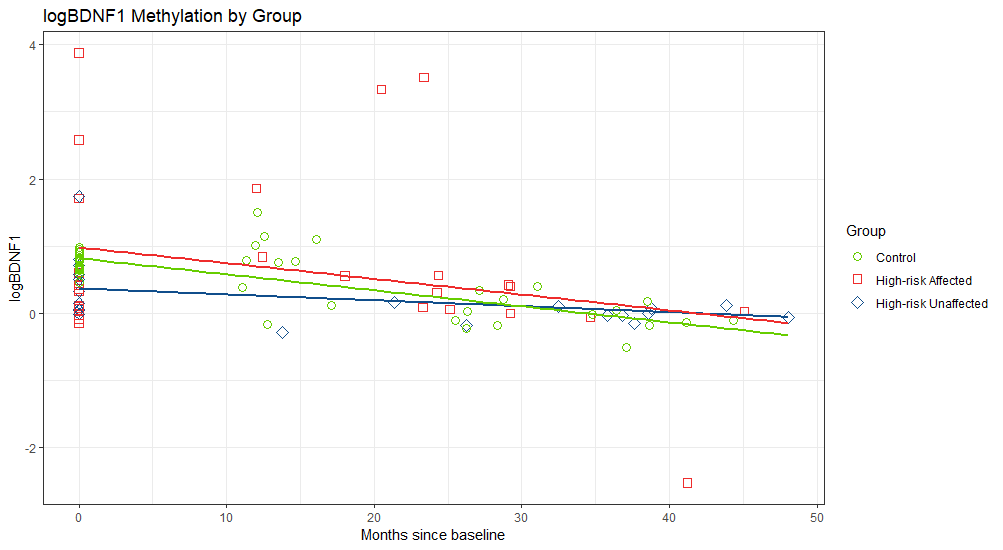


**Figure S5.** Longitudinal Study - *IL1B* methylation profiles


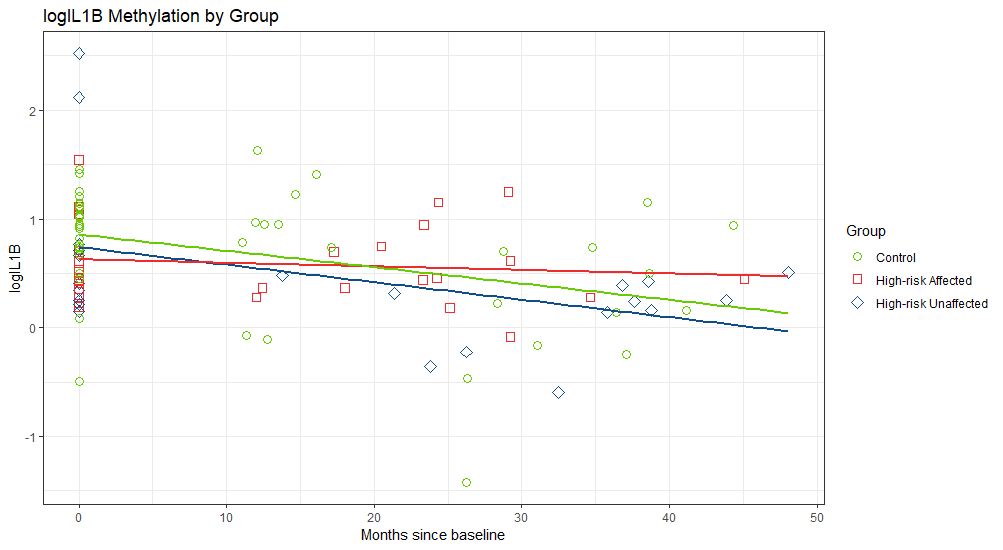


**Figure S6.** Longitudinal Study - *IL6* methylation profiles


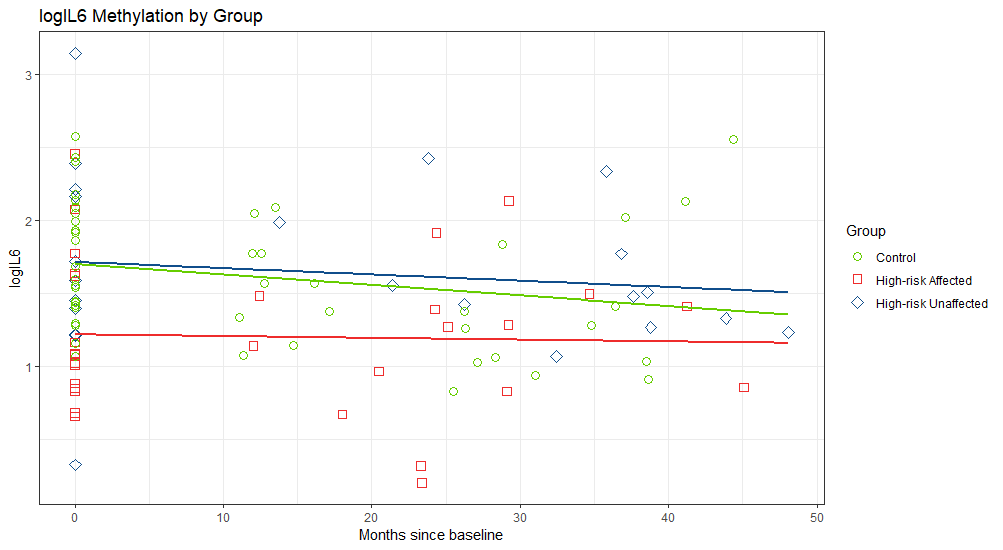

Supplement: Supplementary file 1 — Additional file 1. Supplementary tables and figures. [file 40345_2019_152_MOESM1_ESM.docx]
